# Composition and taste of beef, pork, and duck meat and bioregulatory functions of imidazole dipeptides in meat

**DOI:** 10.1038/s41598-023-29351-z

**Published:** 2023-02-06

**Authors:** Katsuko Kajiya, Madoka Arino, Akari Koshio, Yuji Minami

**Affiliations:** 1 Graduate School of Agriculture, Forestry, and Fisheries, Kagoshima University, Kagoshima, Japan; 2 Department of Food Science and Biotechnology, Faculty of Agriculture, Kagoshima University, 1-21-24 Korimoto, Kagoshima, 890-0065 Japan; 3 The United Graduate School of Agricultural Sciences, Kagoshima University, Kagoshima, Japan

**Keywords:** Biochemistry, Chemical biology

## Abstract

This study quantified the nutritional components and imidazole dipeptide levels of commercially available meats (beef, pork, and duck), and their effects on taste were quantified via taste recognition devices. Although meat and its products are considered high-risk diets, meat components, such as imidazole dipeptides, exert bioregulatory functions. Further, considering their bioregulatory function, commercial meats’ antioxidant activity and vascular endothelial function were examined. Characteristic variations in nutritional components were observed depending on the type and part of meat analyzed. These components affected the taste and texture of meat. The main imidazole dipeptides detected were anserine (duck meat) and carnosine (beef and pork). Meat with larger quantities of total imidazole dipeptide demonstrated better sensory test results. Therefore, anserine and carnosine effects on taste were determined using a taste recognition device; carnosine alone produced a noticeably bitter taste, whereas adding anserine reduced bitterness and enhanced umami taste. In a few cases, cooking enhanced the quantity of carnosine and/or anserine and their antioxidant activities. We demonstrated the ability of imidazole dipeptides, particularly anserine, to improve nitric oxide production in vascular endothelial cells. This study provides essential information for health-conscious consumers to develop high-quality, functional meat products.

## Introduction

Japan boasts the world's highest life expectancy due to advancements in medicine, public health, and diet. One significant change in the Japanese diet over the last 70 years has been the rapid increase in the consumption of food sourced from animals, such as meat, milk, and eggs. In particular, meat is rich in high-quality proteins, has a high amino acid score, and is widely consumed by individuals of all ages. Furthermore, people are expected to live longer and healthier lives to counter the ever-advancing average age of society. However, as people age, there is an increase in the rate of various diseases, such as lifestyle-related diseases and dementia. Against this backdrop, research into improving brain and blood vessel function through daily dietary intake is attracting attention. Studies using cultured neuronal cells have shown that taurine can improve the function of the brain and blood vessels. Studies using cultured nerve cells have revealed that taurine^1^ and GABA (gamma-aminobutyric acid)^2^ have neuroprotective effects, and curcumin has an inhibitory effect on senile plaque aggregation^3^. Although research on the functionalities of meat and its products remains limited, imidazole dipeptide is known to be one of the major functional compounds in meat^4^. Imidazole dipeptide, a generic name for dipeptides consisting of histidine-linked dipeptides containing an imidazole group, is abundant in meat, especially muscle and brain. The best-known imidazole dipeptides in the skeletal muscle are carnosine (β-alaninyl-L-histidine) and anserine (β-alanyl-N-methylhistidine)^5^. These two molecules have antioxidant properties, suppressing protein degradation induced by reactive oxygen generated via neutrophils^6–8^. They also confer anti-fatigue properties because they inhibit the pH drop induced by the lactic acid produced in muscles during exercise^9,10^. More recently, analysis of Alzheimer's disease using mouse models^11^ and the results of studies on middle-aged and older human volunteers have shown that imidazole dipeptide is effective in improving memory function^12^. However, an abundance of functional compunds is not necessarily accompanied by good taste. For example, catechins in green tea and resveratrol in wine are functional compunds that are expected to have high biological regulatory functions; however, both are sources of astringency and bitterness^13^. An abundance of health-functional ingredients is meaningless if they detract from the taste of the meat. Therefore, we investigated the content of imidazole dipeptide as a functional component in addition to the general nutritional components of commercially available cuts of meat (beef, pork, duck) to determine its effect on the taste of commercial meats. Despite extensive sensory evaluations of meat^14,15^, no comparative studies have been conducted on the relationship between chemical taste and nutritional and functional components. This study therefore aimed to clarify the relationship between objectively quantified taste and health functionality using antioxidant activity and vascular endothelial function and to provide basic data for using commercial meat in a healthy diet. Overall, these findings increase the range of options available to health-conscious consumers and to professional researchers interested in developing new functional meat products.

## Materials and methods

### Chemicals

Pancreatin, ethanol, hexane, dichloromethane, L-anserine, L-carnosine, and 2,2-diphenyl-1-picrylhydrazyl (DPPH) were obtained from Fujifilm Wako Chemical Corporation (Osaka, Japan). 2,3-diaminonaphthalene (DAN) was purchased from Dojindo Laboratories (Kumamoto, Japan). High-performance liquid chromatography (HPLC) grade pepsin and acetonitrile were purchased from Sigma-Aldrich (St. Louis, MO, USA). Porcine vascular endothelial cells (VECs) derived from aortic arteries were obtained from Cosmo Bio Co., Ltd. (Tokyo, Japan). All meat samples were purchased from the general sections of local supermarkets. We examined beef, pork, and duck meat produced in Kagoshima, Japan, and their imported counterparts. These were selected from among the best-selling products in supermarkets: beef loin from the USA, beef round from Australia, pork from Canada, and duck from Thailand. All these meats are frozen once for distribution, then thawed and sold within 12 h at 2 °C. They were then stored at 2 °C for up to 30 min before further experiments. Three samples were purchased on different days to eliminate individual differences. The fat was trimmed before study, and the meat was thinly sliced.

### Artificial digestion

Meat is generally consumed after cooking. Therefore, we included half raw meat samples and another half subjected to the same heat treatment after weight alignment. Each meat sample (0.5 g) was pulverized, heated in a microwave oven (500 W) for approximately 3 min, and then subjected to artificial digestion^16,17^, to simulate the cooking and digestion processes, respectively. After adding artificial gastric juice (pepsin, 20 mg; 0.1 M HCl, 2 mL; water, 8 mL), the mixture was stirred for 4 h using a shaking incubator (220 rpm, 40 °C; Eppendorf, Hamburg, Germany). Next, the mixture pH was adjusted to approximately 8.5 (the optimal pH for pancreatin function) using 1 M NaOH. Thereafter, 1 mL of 50 mg/mL pancreatin was added as the artificial intestinal fluid, and the mixture was stirred for 4 h under the same conditions. The pH was then measured every hour and maintained between 8.0 and 10.0. After the artificial digestion reaction was terminated by deactivating the enzyme in boiling water, the pH of the solution was adjusted to neutral. Finally, each solution was freeze-dried using a freeze-dryer (FDU-1200; Tokyo Rikakikai Co., Ltd, Tokyo, Japan) and stored below –20 °C.

### Nutrient analysis

Analyses of water content (normal pressure heating and drying method)^18^, protein (Kjeldahl method)^19^, fat (Soxhlet extraction method)^20^, ash (dry ashing method)^21^, vitamins (HPLC)^22^, and fatty acids (gas chromatography)^23^ were outsourced to Japan Food Research Laboratories (Fukuoka, Japan), which specializes in the analysis of general nutritional components.

### Quantification of imidazole dipeptides

The freeze-dried sample was passed through a 0.45-μm disk filter (13 HP; Advantech Toyo Co., Ltd., Tokyo, Japan) and analyzed using HPLC (LC2000 series and Extrema; JASCO Corporation, Tokyo, Japan)^24–26^. Individual compounds were identified based on their retention times. Highly selective spectral data were generated using ultraviolet–visible adsorption detectors (UV-2070 and UV-4075; JASCO), a photodiode array detector (MD-4010; JASCO), and a mass spectrometer (3200QTRAP; SCIEX, Framingham, MA, USA). A TSKgel ODS-100Z column (particle diameter 5 μm, 4.6 mm × 25 cm, Tosoh Corporation; Yamaguchi, Japan) was used. The column temperature and flow rate were 40 °C and 0.8 mL/min, respectively. The isocratic mobile phase comprised 200 mM ammonium dihydrogen phosphate, 100 mM sodium pentanesulfonate, and 4% acetonitrile (pH adjusted to 2.0 with HCl). Imidazole dipeptides were detected at 220 nm, and anserine and carnosine retention times were approximately 9.5 and 12 min, respectively.

### Sensory evaluation and taste measurement

Cooked meat samples (beef, pork, and duck) were comprehensively evaluated for the following sensory attributes: sweetness, bitterness, hardness, and umami. Each item was rated on a five-point scale, with scores ranging between + 2 and – 2^27^. Higher scores reflect sweeter and stronger umami tastes, less bitterness, and more tenderness. Each sample also received an overall score. The evaluation was performed by 19 trained panelists at Kagoshima University, Kagoshima, Japan.

The cooked meat samples (beef, pork, and duck) and distilled water (20 mg/mL) were mixed using a magnetic stirrer (SR 100, Advantec; Japan) for 30 min. The mixture was then sonicated (ASU-6M; AS ONE Corporation, Osaka, Japan) for 15 min, followed by centrifugation at 4 °C and 1600×*g* for 10 min (RX-200; Tomy, Tokyo, Japan), and the supernatant was collected. The supernatant was again centrifuged, and the resulting aliquot was tested using a taste recognition device (TS-5000Z; Intelligent Sensor Technology, Inc., Kanagawa, Japan)^28^. Standard solutions (anserine and carnosine) were prepared in distilled water to the desired concentration. The sensors used in the device were pre-conditioned with an internal solution (3.33 M potassium chloride and saturated silver chloride) and a reference solution for 24 h. The sensors and reference electrodes were attached to the device and calibrated accordingly. The sample solution was then placed in a special cup, and the initial taste (sourness, saltiness, umami, acidic bitterness, astringency, and sweetness) and aftertaste (acidic bitterness, astringency, richness, basic bitterness, and hydrochloride salts) were measured.

### Antioxidant activity measurement

The DPPH method measured the antioxidant activities of hydrophilic and lipophilic extracts^29,30^. In summary, 50 mg of the freeze-dried sample was placed in a microcentrifuge tube, and 2 mL of the extraction solution was added. A methanol/water/acetic acid (90/9.5/0.5, v/v/v) solution was used for hydrophilic extraction, and hexane/dichloromethane (50/50, v/v) was used for lipophilic extraction. The mixture was vortexed, treated ultrasonically for 10 min, and centrifuged at 1600×*g* at 4 °C for 10 min. One milliliter of each supernatant was vacuum-concentrated (VEC-260; Iwaki, Tokyo, Japan), and 1 mL of 50% ethanol was added to each concentrated sample. The samples were then vortexed and ultrasonicated until the concentrate dissolved. The sample solution was then added to a 96-well microplate (50 μL/well), and 50% ethanol was added to each well to make the volume up to 100 μL. Subsequently, 50 μL of 800 μM DPPH solution was added to the sample solution, and the plate was incubated at 25 °C for 20 min in the dark. Absorbance was measured at 540 nm using a microplate reader (Infinite 200 PRO; Tecan, Männedorf, Switzerland). A calibration curve with a correlation coefficient (R^2^) of 0.9983 was generated using Trolox (Sigma-Aldrich) as the reference standard. Each experiment was conducted in quadruplicate. The DPPH scavenging activity was expressed as Trolox equivalents.

### Quantification of nitric oxide (NO) production using a modified Griess method

VECs produce NO, which regulates vasorelaxation and prevents endothelial adherence. To quantify the effect of meat samples on NO production, samples were processed as previously described. NO has a short half-life and is hydrolyzed to NO_2_^−^ and NO_3_^−^. NO_2_^−^ content is measured using the Griess method^31–33^, reducing NO_3_^−^ to NO_2_^−^ using NO_3_^−^ reductase, and measurement of the total NO_2_^−^ concentration. Therefore, the volume of NO is measured indirectly. DAN fluorescence assay, an NO_2_^-^ assay with higher sensitivity than the Griess method, was developed^34^. NO_2_^−^ reacts with DAN under acidic conditions to form a fluorescent naphthalene triazole adduct. We quantified the product by measuring fluorescence intensity using a microplate reader. VECs were seeded in 96-well plates at 5.0 × 10^4^ cells/mL in HuMedia-EG2 (Kurabo, Osaka, Japan) supplemented with 2% fetal bovine serum and cultured in 5% CO_2_ incubator (MCO-5AC; Panasonic, Osaka, Japan). When the cells reached 80% confluence, they were incubated in a medium (100 μL) with or without meat extract (15 μg/mL) for an additional 12 h. The cultured supernatants were then collected by centrifugation at 1000×*g* and 25 °C for 15 min and reduced with nitrate reductase and the respective enzyme cofactors (iron, molybdenum, and cytochrome) at 37 °C for 30 min, followed by incubation for 15 min with DAN. Fluorescence intensity was measured at 450 nm under 360 nm excitation using a microplate reader. The concentration of NO per sample was calculated via a calibration curve (R^2^ = 0.9905) obtained using an NaNO_2_ standard solution. The results were normalized to the controls.

### Statistical analyses

Significant differences among groups were assessed using the Student’s *t*-test. Nine repeat experiments were carried out, with n = 3 for each repeat experiment performed with meat purchased on another 3 days. Data are presented as the mean ± standard deviation. *P* < 0.05 was considered statistically significant^27^.

## Results

### Nutrition analysis

We found no significant differences between the nutritional components of raw and cooked beef cuts (Tables 1 and 2). Similar to data with beef, pork and duck data also showed no significant differences in nutrient composition between raw and cooked meat (data not shown). Hence, the data for pork and duck samples in Tables 1 and 2 only include meat that has been cooked to the point at which it is intended to be eaten. Japanese Black cattle meat is marbled meat, characterized by high-fat content. The findings demonstrated that Kagoshima beef contained 20–33% less moisture, 13–33% less protein, and 6.7–10.9-fold more fat than imported beef. The Kagoshima loin cut had a lower protein (22–33%) and higher fat (1.7–1.9-fold) content than did the Kagoshima round cut of beef. In contrast, the loin and round cuts of imported beef demonstrated similar protein contents and higher fat (2.3-fold) in the loin. Compared to pork loin and tenderloin, pork belly was generally low in water (42–43% and 26–30% less moisture than Kagoshima pork and imported meat, respectively), low in protein (Kagoshima pork down 49–54% and 24–30% lower protein than Kagoshima pork and imported meat, respectively), and high in fat (Kagoshima pork up 16-fold and 4.5–22-fold higher fat than Kagoshima pork and imported meat, respectively). For duck meat samples, no difference was found between the parts (thigh or breast) and between the local and imported meat. The ash content was similar in all meat samples. The vitamin B1 (VB1) and B2 (VB2) content decreased as follows: pork > duck > beef, and duck > pork ≈ beef, respectively (Table 1). However, no significant differences were found for other vitamins (data not shown).

**Table 1:** Water, protein, fat, ash, VB1, and VB2 contents in the 100-g fresh meat samples.

	Type	Part	State	Water (g)		Protein (g)		Fat (g)		Ash (g)		VB1 (mg)		VB2 (mg)	
Beef	Kagoshima	Loin	Raw	48.8 ± 0.3		12.8 ± 0.1		36.4 ± 0.0		0.7 ± 0.0		0.06 ± 0.00		0.16 ± 0.00	
			Cooked	51.1 ± 0.4		13.4 ± 0.1		32.7 ± 0.1		0.7 ± 0.0		0.06 ± 0.00		0.17 ± 0.00	
		Round	Raw	60.9 ± 0.2		16.9 ± 0.0		18.8 ± 0.4		0.9 ± 0.0		0.09 ± 0.00		0.18 ± 0.01	
			Cooked	60.9 ± 0.0	***	17.0 ± 0.0	***	19.6 ± 0.0	***	0.9 ± 0.1	***	0.09 ± 0.00	***	0.18 ± 0.00	***
	Imported	Loin	Raw	73.0 ± 0.2		19.1 ± 0.1		5.4 ± 0.1		1.0 ± 0.1		0.09 ± 0.00		0.21 ± 0.01	
			Cooked	73.0 ± 0.1		19.5 ± 0.0		4.2 ± 0.2		1.0 ± 0.0		0.09 ± 0.00		0.21 ± 0.01	
		Round	Raw	75.7 ± 0.1		19.3 ± 0.0		2.1 ± 0.1		1.1 ± 0.0		0.10 ± 0.00		0.20 ± 0.00	
			Cooked	75.3 ± 0.0	***	19.8 ± 0.0	**	1.8 ± 0.1	***	1.0 ± 0.0		0.10 ± 0.00	***	0.21 ± 0.01	
Pork	Kagoshima	Loin	Cooked	73.0 ± 0.1		21.6 ± 0.1		2.9 ± 0.2		1.1 ± 0.0		1.32 ± 0.01		0.18 ± 0.00	
		Belly	Cooked	42.4 ± 0.3	***	10.6 ± 0.0	***	46.9 ± 0.1	***	0.6 ± 0.0	***	0.52 ± 0.01	***	0.11 ± 0.00	***
		Tenderloin	Cooked	74.7 ± 0.0	***	19.6 ± 0.0	***	2.9 ± 0.1		1.1 ± 0.1		1.70 ± 0.02	***	0.33 ± 0.01	***
	Imported	Loin	Cooked	71.8 ± 0.3		18.5 ± 0.0		7.2 ± 0.1		1.1 ± 0.0		0.75 ± 0.01		0.27 ± 0.00	
		Belly	Cooked	52.8 ± 0.2	***	14.1 ± 0.0	***	32.3 ± 0.2	***	0.7 ± 0.1	**	0.66 ± 0.01	***	0.15 ± 0.00	***
		Tenderloin	Cooked	75.7 ± 0.1	***	20.1 ± 0.0	***	1.5 ± 0.0	***	1.2 ± 0.0	***	1.32 ± 0.01	***	0.26 ± 0.00	*
Duck	Kagoshima	Thigh	Cooked	74.0 ± 0.1		20.4 ± 0.0		2.7 ± 0.2		1.2 ± 0.1		0.27 ± 0.00		0.35 ± 0.01	
		Breast	Cooked	74.5 ± 0.1	***	20.5 ± 0.0		2.1 ± 0.1	**	1.2 ± 0.0		0.36 ± 0.00	***	0.53 ± 0.01	***
	Imported	Thigh	Cooked	75.1 ± 0.1		18.8 ± 0.1		3.3 ± 0.0		1.1 ± 0.0		0.31 ± 0.00		0.37 ± 0.01	
		Breast	Cooked	75.2 ± 0.1		19.8 ± 0.1	**	2.2 ± 0.0	***	1.3 ± 0.0	***	0.44 ± 0.00	***	0.32 ± 0.01	***

*Indicates *P* < 0.05 vs. loin (beef and pork) or thigh (duck). **Indicates *P* < 0.01 vs. loin (beef and pork) or thigh (duck). ***Indicates *P* < 0.001 vs. loin (beef and pork) or thigh (duck).

**Table 2:** Fatty acids in the 100-g fresh meat samples.

	Type	Part	State	Total (g)		Saturated (g)		Unsaturated (g)							
								Mono		Poly		n-3		n-6	
Beef	Kagoshima	Loin	Raw	32.6 ± 0.0		11.70 ± 0.02		20.00 ± 0.02		0.81 ± 0.02		0.07 ± 0.02		0.72 ± 0.02	
			Cooked	29.7 ± 0.0		10.70 ± 0.01		18.30 ± 0.01		0.74 ± 0.02		0.07 ± 0.02		0.67 ± 0.02	
		Round	Raw	16.6 ± 0.0	***	6.25 ± 0.01	***	9.74 ± 0.00	***	0.62 ± 0.02	***	0.05 ± 0.01	**	0.57 ± 0.01	***
			Cooked	17.4 ± 0.0	***	6.54 ± 0.00	***	10.20 ± 0.01	***	0.64 ± 0.02	***	0.05 ± 0.01	**	0.59 ± 0.01	**
	Imported	Loin	Raw	4.4 ± 0.0		1.96 ± 0.01		2.20 ± 0.01		0.24 ± 0.01		0.03 ± 0.00		0.20 ± 0.02	
			Cooked	3.4 ± 0.0		1.50 ± 0.00		1.66 ± 0.00		0.19 ± 0.00		0.02 ± 0.00		0.17 ± 0.01	
		Round	Raw	1.5 ± 0.0	***	0.63 ± 0.01	***	0.74 ± 0.00	***	0.15 ± 0.00	***	0.03 ± 0.00		0.11 ± 0.01	***
			Cooked	1.1 ± 0.0	***	0.46 ± 0.01	***	0.52 ± 0.00	***	0.16 ± 0.00	**	0.03 ± 0.00		0.12 ± 0.01	**
Pork	Kagoshima	Loin	Cooked	2.5 ± 0.0		0.97 ± 0.00		1.31 ± 0.01		0.19 ± 0.00		0.01 ± 0.00		0.17 ± 0.01	
		Belly	Cooked	43.7 ± 0.0	***	18.50 ± 0.04	***	21.80 ± 0.04	***	3.35 ± 0.04	***	0.24 ± 0.03	***	3.11 ± 0.04	***
		Tenderloin	Cooked	2.3 ± 0.0		1.00 ± 0.02		1.03 ± 0.00		0.30 ± 0.00	**	0.02 ± 0.00		0.28 ± 0.02	
	Imported	Loin	Cooked	6.3 ± 0.0		2.53 ± 0.00		2.33 ± 0.01		1.48 ± 0.00		0.12 ± 0.01		1.35 ± 0.03	
		Belly	Cooked	30.0 ± 0.0	***	12.10 ± 0.04	***	14.10 ± 0.04	***	3.85 ± 0.04	***	0.23 ± 0.01	***	3.61 ± 0.04	***
		Tenderloin	Cooked	1.1 ± 0.0		0.43 ± 0.01	***	0.47 ± 0.00		0.23 ± 0.00	***	0.01 ± 0.00		0.22 ± 0.01	
Duck	Kagoshima	Thigh	Cooked	2.1 ± 0.0		0.70 ± 0.01		0.89 ± 0.00		0.55 ± 0.02		0.04 ± 0.00		0.51 ± 0.01	
		Breast	Cooked	1.6 ± 0.0		0.50 ± 0.00		0.59 ± 0.00		0.39 ± 0.01		0.02 ± 0.00		0.37 ± 0.02	
	Imported	Thigh	Cooked	2.1 ± 0.1		0.77 ± 0.01		1.29 ± 0.01	***	0.61 ± 0.02		0.04 ± 0.00		0.57 ± 0.02	
		Breast	Cooked	1.5 ± 0.1		0.51 ± 0.00		0.70 ± 0.00	***	0.31 ± 0.01		0.02 ± 0.00		0.32 ± 0.02	

*Indicates *P* < 0.05 vs. loin (beef and pork) or thigh (duck). **Indicates *P* < 0.01 vs. loin (beef and pork) or thigh (duck). ***Indicates *P* < 0.001 vs. loin (beef and pork) or thigh (duck).

Owing to significant differences in fat content, the fatty acid composition was examined in further detail (Table 2). Overall, there were more unsaturated fatty acids than saturated ones. The individual components of fatty acids were not quantified; however, we expect the components to include myristic acid 14:0, palmitic acid 16:0, and stearic acid 18:0 for saturated fatty acids; and myristoleic acid 14:1, palmitoleic acid 16:1, oleic acid 18:1, linoleic acid 18:2 (n-6), α-linolenic acid 18:3 (n-3), eicosenoic acid 20:1, eicosatrienoic acid 20:3 (n-6), and arachidonic acid 20:4 (n-6) for unsaturated fatty acids. The amount of unsaturated fatty acids in total fatty acids were as follows: Kagoshima beef loin (64%), Kagoshima beef round (62%), imported beef loin (54%), imported beef round (62%), Kagoshima pork loin (60%), Kagoshima pork belly (58%), Kagoshima pork tenderloin (58%), imported pork loin (60%), imported pork tenderloin (64%), Kagoshima duck (60%), Kagoshima pork belly (58%), Kagoshima pork tenderloin (58%), imported pork loin (60%), imported pork belly (60%), imported pork tenderloin (64%), Kagoshima duck (69%), Kagoshima duck thigh (69%), Kagoshima duck breast (61%), imported duck thigh (90%), and imported duck breast (67%). Most unsaturated fatty acids were monounsaturated, with the highest percentage found in beef. Monounsaturated fatty acids in total unsaturated fatty acids were as follows: Kagoshima beef loin (96%), Kagoshima beef round (94%), imported beef loin (90%), imported beef round (76%), Kagoshima pork loin (87%), Kagoshima pork belly (87%), Kagoshima pork tenderloin (77%), imported pork loin (61%), imported pork belly (79%), imported pork tenderloin (67%), Kagoshima duck thigh (62%), Kagoshima duck breast (60%), imported duck thigh (68%), and imported duck breast (69%). Polyunsaturated fatty acids (n-3 and n-6) were also found, with the n-6 type being more common. The n-6 fatty acids in total polyunsaturated fatty acids were as follows: Kagoshima beef loin (91%), Kagoshima beef round (92%), imported beef loin (90%), imported beef round (89%), Kagoshima pork loin (75%), Kagoshima pork belly (93%), Kagoshima pork tenderloin (93%), imported pork loin (91%), imported pork belly (94%), imported pork tenderloin (96%), Kagoshima duck thigh (93%), Kagoshima duck breast (95%), imported duck thigh (93%), and imported duck breast (99%). In particular, duck meat is considered a source of high-quality polyunsaturated fatty acids, with n-6 fatty acids accounting for 20% or more of the total. The n-6 fatty acids in total unsaturated fatty acids were as follows: Kagoshima beef loin (3%), Kagoshima beef round (5%), imported beef loin (8%), imported beef round (14%), Kagoshima pork loin (10%), Kagoshima pork belly (11%), Kagoshima pork tenderloin (17%), imported pork loin (26%), imported pork belly (17%), imported pork tenderloin (24%), Kagoshima duck thigh (26%), Kagoshima duck breast (27%), imported duck thigh (23%), and imported duck breast (24%). The total polyunsaturated fatty acid content was higher in Kagoshima beef than in imported beef (three to fourfold). Pork belly contained the highest saturated and unsaturated fatty acid among all samples.

### Quantification of imidazole dipeptides

**Figure 1:**
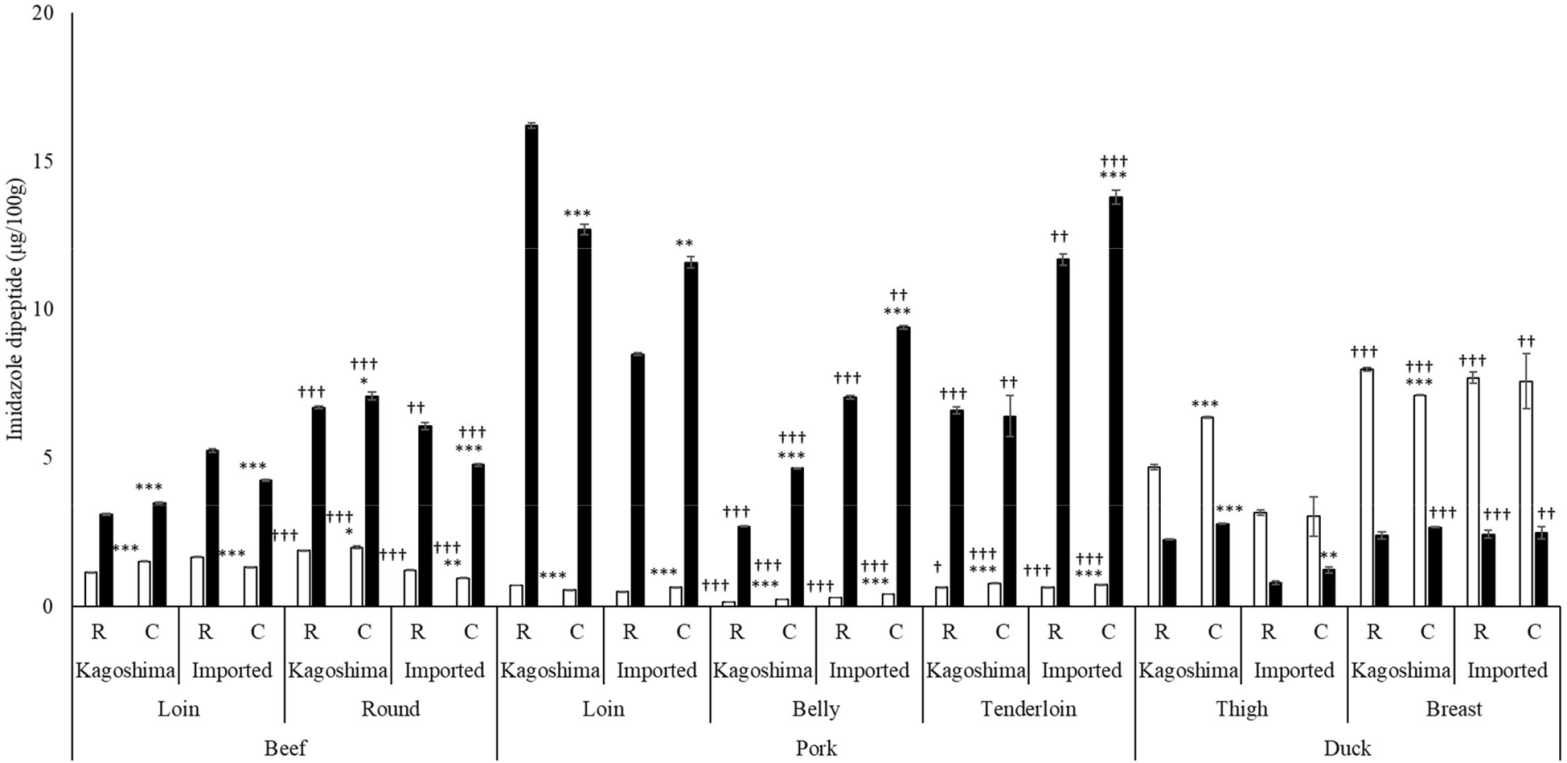
Comparison of imidazole dipeptides in raw and cooked meat extracts. *R* raw, *C* cooked. Anserine: white bars, carnosine: black bars. Asterisk indicates *P* < 0.05 vs. raw. Double asterisk indicates *P* < 0.01 vs. raw. Triple asterisk indicates *P* < 0.001 vs. raw. Dagger indicates *P* < 0.05 *vs*. loin (beef and pork) or thigh (duck). Double dagger indicates *P* < 0.01 vs. loin (beef and pork) or thigh (duck). Triple dagger indicates *P* < 0.001 vs. loin (beef and pork) or thigh (duck).

Anserine and carnosine calibration curves with R2 0.999 were obtained for cooked and raw beef. As shown in Fig. 1, beef contained more carnosine than anserine (2.3–5.0-fold), with 1.1–2.1-fold higher carnosine in the round cut than in the loin cut. The carnosine level was higher in the Kagoshima beef round cut than in the imported cut (1.5-fold). However, the carnosine level was higher in the imported loin cut than in the Kagoshima loin cut (1.2–1.7-fold). Pork also contained more carnosine than anserine (8.1–23.0-fold), higher than did beef and duck meat. The carnosine level was higher in the loin of Kagoshima pork (1.1–1.9-fold), belly (2.0–2.6-fold), and tenderloin (1.8–2.2-fold) cuts of imported pork. In contrast, duck meat contained higher anserine than carnosine (2.1–4.0-fold), which was higher in duck meat than in beef (1.5–4.0-fold) and pork (3.8–10.1-fold). The duck breast had a higher anserine concentration than did the thigh (1.1–2.5-fold). Kagoshima duck thigh contained more anserine than did the imported duck thigh (1.5–2.1-fold). Moreover, cooking increased the levels of several imidazole dipeptides (in Kagoshima beef loin and round, Kagoshima pork belly, Kagoshima duck thigh, and imported pork loin, belly, and tenderloin).

### Taste measurement

All samples received the lowest score for "bitterness," indicating no bitterness in the meat. For the overall score of beef samples, Kagoshima round cut received the highest total sensory evaluation score, whereas the imported round cut received the lowest score (Table 3). This was attributed to the Kagoshima round cuts having the highest sweetness and umami and the imported roundcuts having the lowest. Among all pork samples, despite having the lowest sweetness and umami scores, imported tenderloin had the highest overall score, whereas Kagoshima tenderloin had the lowest overall score (Table 3). This indicates that the texture of pork affects the taste, as the imported tenderloin with the least hardness (soft) had the highest overall score and the Kagoshima tenderloin with the lowest hardness had the lowest overall score. Kagoshima duck breast and thigh received equally high scores, whereas imported thigh received the lowest score among all duck samples (Table 3). This was also influenced by the high scores for sweetness and umami flavors in the overall score.

**Table 3:** Overall sensory evaluation of cooked meat.

	Part	Type	Overall score	Sweetness	Bitterness	Hardness	Umami flavors
Beef^a^	Loin	Kagoshima	+ 0.9	+ 1.4	–2	+ 1.2	+ 2.0
		Imported	+ 1.0	+ 1.1	–2	+ 1.8	+ 1.5
	Round	Kagoshima	+ 2.0	+ 1.6	–2	+ 1.7	+ 2.0
		Imported	+ 0.8	+ 0.9	–2	+ 1.3	+ 1.4
Pork^a^	Loin	Kagoshima	+ 1.8	+ 1.3	–2	+ 1.6	+ 1.4
		Imported	+ 1.5	+ 1.1	–2	+ 1.4	+ 1.5
	Belly	Kagoshima	+ 1.1	+ 1.8	–2	+ 1.1	+ 1.5
		Imported	+ 1.2	+ 1.9	–2	+ 1.3	+ 1.3
	Tenderloin	Kagoshima	+ 1.0	+ 1.6	–2	+ 1.0	+ 1.4
		Imported	+ 2.0	+ 0.8	–2	+ 1.8	+ 0.9
Duck^a^	Thigh	Kagoshima	+ 1.9	+ 1.7	–2	+ 1.8	+ 1.0
		Imported	+ 1.1	+ 1.2	–2	+ 1.5	+ 0.4
	Breast	Kagoshima	+ 1.8	+ 1.8	–2	+ 1.7	+ 0.8
		Imported	+ 2.0	+ 1.3	–2	+ 1.9	+ 0.5

^a^Cooked meat samples (beef, pork, and duck) were comprehensively evaluated for sweetness, bitterness, hardness, and umami flavors using sensory evaluation. Each item was rated on a five-point scale, ranging from + 2 to –2. A higher score indicated better overall sensory quality. Samples were evaluated by 19 trained panelists.

**Figure 2:**
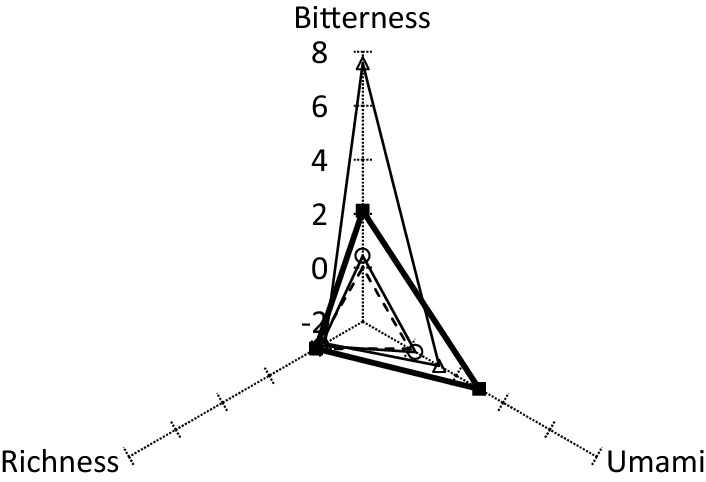
Distribution of bitterness, richness, and umami contents obtained using a taste recognition device. Values are shown relative to the control (dotted line). White circles indicate anserine only; white triangles indicate carnosine only; black squares indicate anserine and carnosine.

A taste recognition device estimated the bitterness, richness, and umami flavors of anserine and carnosine standards with a high degree of confidence. Therefore, we focused on imidazole dipeptide content (Fig. 1) and its taste, using Kagoshima pork loin with a typical imidazole dipeptide ratio as a representative example. Bitterness of carnosine alone (7.57) was much more bitter than that of anserine alone (0.44) (Fig. 2). However, when taste recognition measurements were performed by reproducing the ratio of anserine and carnosine, bitterness was reduced by less than half (7.57 to 2.11) and umami increased more than twice (0.25 to 2.98). Therefore, the coexistence of anserine and carnosine reduced the bitterness of the meat and increased the umami level.

### Antioxidant activity

**Figure 3:**
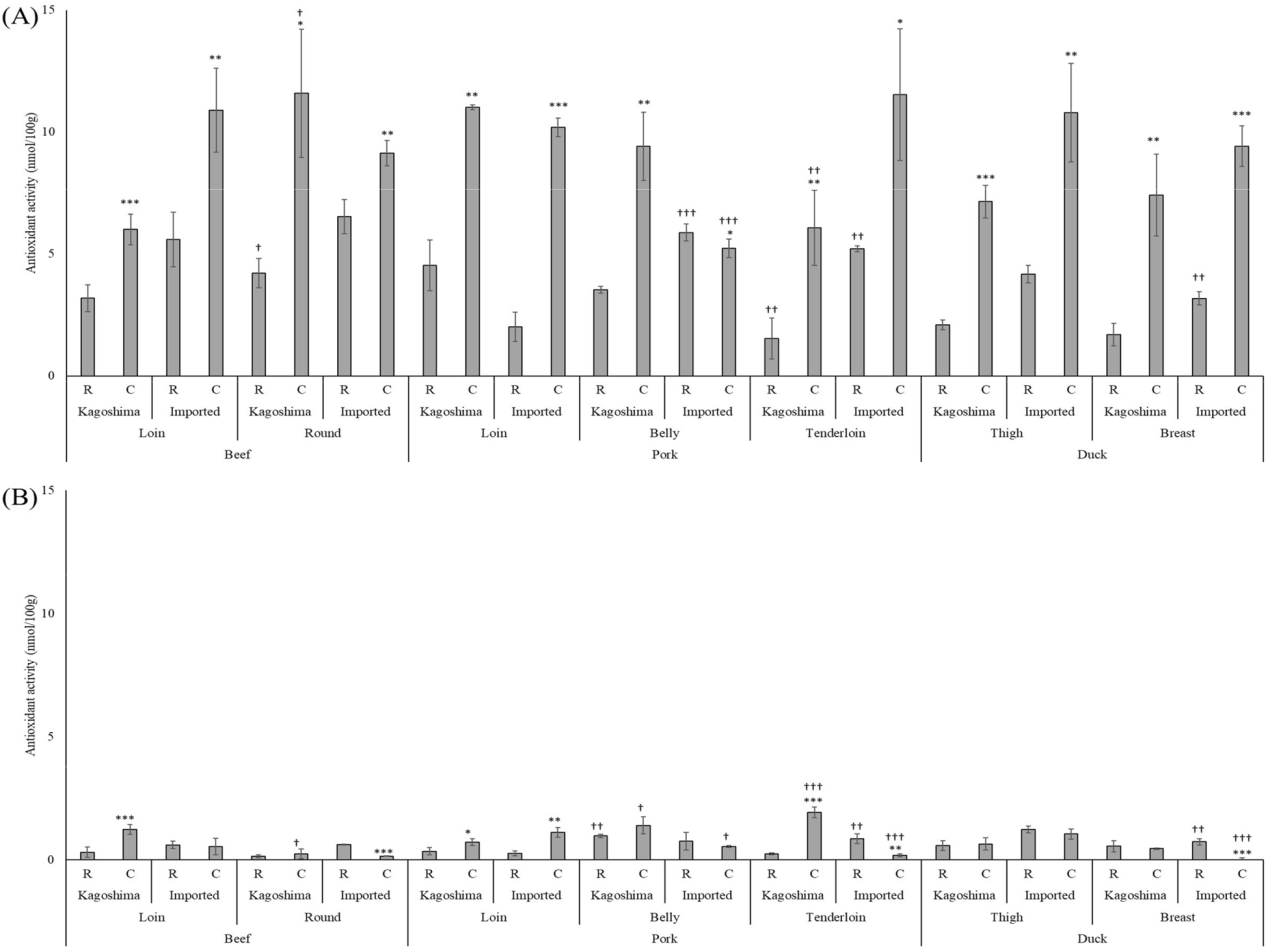
Antioxidant activities of (**A**) hydrophilic and (**B**) lipophilic meat-sample extracts. *R* raw, *C* cooked. Asterisk indicates *P* < 0.05 *vs*. raw. Double asterisk indicates *P* < 0.01 vs. raw. Triple asterisk indicates *P* < 0.001 vs. raw. Dagger indicates *P* < 0.05 vs. loin (beef and pork) or thigh (duck). Double dagger indicates *P* < 0.01 vs. loin (beef and pork) or thigh (duck). Triple dagger indicates *P* < 0.001 vs. loin (beef and pork) or thigh (duck).

In all domestic and imported meat samples, hydrophilic extracts demonstrated substantial antioxidant activity (Fig. 3A), whereas the lipophilic extracts did not (Fig. 3B), with differences ranging from 3.0- to 61.7-fold. Further, there is a distinct difference between the antioxidant activity of cooked and raw meat, as evident from Fig. 3A, except for the imported pork belly. The antioxidant activity of the standard anserine was concentration-dependent (R^2^ = 0.7815) and reached a plateau at 1.25 mg/mL. Similarly, the standard carnosine sample showed concentration-dependent antioxidant activity (R^2^ = 0.8416), which reached its peak at 2.5 mg/mL.

### Endothelial function

**Figure 4:**
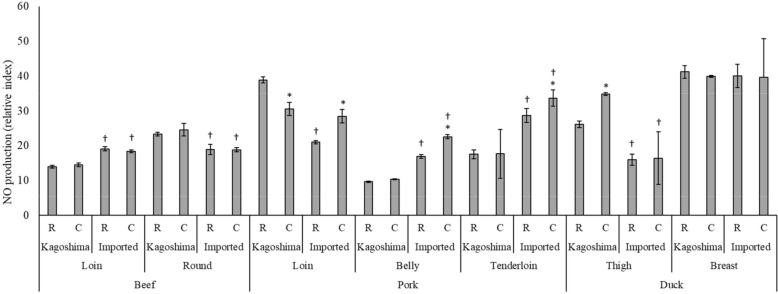
Effect of meat extract on nitric oxide production via vascular endothelial cells. *R* raw, *C* cooked. Asterisk indicates *P* < 0.05 vs. raw. Dagger indicates *P* < 0.05 vs. Kagoshima meat.

VECs produce NO in their normal state. However, aging and stress can limit this capacity. Therefore, foods with enhanced NO production are healthier. Figure 4 shows the effect of the meat extracts on NO production by VECs in vitro; the ability to improve NO production was confirmed in all meats. Duck meat extracts, particularly raw and cooked breast, produce high NO among all others. Notably, when using a standard reagent, 1.5 ng/mL carnosine has an NO production index of only 2.2, whereas 1.5 ng/mL anserine is 4.5.

## Discussion

In this study, we investigated the nutritional components and bioregulatory functions of retail meat cuts from Japanese Black cattle, Black Berkshire pigs, and Satsuma Black Aigamo ducks produced in Kagoshima, Japan, and then compared the results with those from imported meat. Water, protein, fat (including fatty acid), VB1, and VB2 contents varied depending on the type and part of meat analyzed. However, the ash and other vitamin content remained relatively unchanged. Imidazole dipeptides were found in the extracts of raw and cooked duck meat (mostly anserine) and raw and cooked beef and pork (mostly carnosine). For the same cut, the imidazole dipeptide levels were higher in the locally produced meat than in the imported meat. We investigated the correlation between imidazole dipeptide content and each nutrient, although no correlation was found. Cooked meat is expected to contain lower levels of imidazole dipeptides than raw meat, owing to the influence of pyrolysis, hydrolysis, and the Maillard reaction. However, in a few cases, these levels were higher after cooking. The amino acid content in some vegetables and meat was reported to increase during broiling, roasting, or vacuum cooking, as protein degradation proceeded^35–38^. Furthermore, a high correlation was observed between total imidazole dipeptide content and sensory evaluation in meat, with R^2^ values of 0.9872, 0.8224, and 0.9526 for beef, pork, and duck samples, respectively. Moreover, amino acid levels in different parts of the animal did not differ significantly, and the imidazole content ratio has a significant effect on taste. Therefore, when the taste of carnosine and anserine was measured individually, carnosine alone produced a pronounced bitter taste. However, the addition of anserine was found to decrease bitterness and increase umami. This was attributed to the fact that anserine had a masking effect on bitterness, and umami was attributed to the synergistic effect of anserine and carnosine. Although anserine and ginsenosides are reported to have synergistic effects on uric acid excretion^39^, we provide the first evidence regarding the synergistic effects of anserine and carnosine on taste.

Proteins are broken down in the gastrointestinal tract when meat is eaten; hence, even if the meat contains high levels of imidazole dipeptide, the protein may be digested and not function. Therefore, we performed an artificial digestion process that mimics human digestion and compared component quantification and biological regulatory function in a state similar to that of naturally absorbed components. The hydrophilic meat extracts demonstrated higher antioxidant activity than did the lipophilic extracts. Further, all meat samples exhibited higher antioxidant activities after cooking, except for the imported pork belly. This was attributed to the enhanced component extraction efficiency after cooking. Raw imported pork belly is reported to have high antioxidant activity, owing to its high lipoic acid, L-carnitine, and glutathione, which may have decreased after cooking because of their heat sensitivity^40^. No consistent trend was observed when the antioxidant activity of Kagoshima meat was compared with that of imported meat. We also examined the antioxidant activity of the standard reagents, anserine and carnosine, and found that each showed antioxidant activity. The antioxidant activity exhibited by the meat was attributed to imidazole dipeptide. This study demonstrated the ability of imidazole dipeptides, particularly anserine, to increase in vitro NO production. Duck meat extracts with high anserine levels showed strong effects on NO production, suggesting that anserine has a more significant impact on NO production than does carnosine. However, NO production was found to be positively correlated with not only the anserine content but also the amount of total imidazole dipeptide (R^2^ = 0.9867). Overall, our results revealed that meat extracts promote NO production through the synergistic action of anserine and carnosine. Considering the available information regarding the antioxidant activity and vascular endothelial function of imidazole dipeptides, we concluded that cooking meat enhances bioregulatory functions and improves its safety and taste. These findings lead to the development of improved meat processing and cooking procedures that boost antioxidant activity and NO generation capability. However, this study was limited to a small number of supermarket samples. Therefore, large-scale research is required to establish the bioregulatory functions and health benefits of different types of meat.

## Conclusion

The main imidazole dipeptides found in the analyzed meat samples were anserine in duck meat and carnosine in beef and pork. These meat samples also showed high antioxidant activity. In a few cases, the imidazole content and antioxidant effects were enhanced via cooking, indicating that cooking provides health benefits in addition to enhancing the taste, aroma, digestibility, and safety of meat. In contrast, the lipophilic extracts exhibited non-significant antioxidant activity, supporting the general dietary recommendation for consuming low-fat meat over high-fat meat. Furthermore, we demonstrated the ability of imidazole dipeptides, particularly anserine, to enhance NO production in VECs. In an aging society, lifestyle-related disease prevention is essential, with an aim to increase healthy life expectancy allowing older adults to live independently without nursing care or medical treatment. This study suggests that imidazole dipeptide in livestock meat may improve antioxidant activity and vascular endothelial function in vivo and can contribute as a functional ingredient in lifestyle-related disease prevention foods. This research provides important information for health-conscious consumers and for developing high-quality functional meat products.

## Data Availability

All data generated or analyzed during this study are included in this published article.
